# Quality of life of people living with chronic hepatitis B: The role of social support system

**DOI:** 10.1371/journal.pgph.0003103

**Published:** 2024-04-26

**Authors:** Yasmin Ibrahim, Beatrice Zovich, Bright Ansah, Catherine Freeland, Michaela Jackson, Thomas Tu, Chari Cohen

**Affiliations:** 1 Hepatitis B Foundation, Doylestown, Pennsylvania, United States of America; 2 Storr Liver Centre, Westmead Clinical School and Westmead Institute for Medical Research, Faculty of Medicine and Health, The University of Sydney, Westmead, Australia; 3 Sydney Institute for Infectious Diseases, University of Sydney at Westmead Hospital, Westmead, Australia; 4 HepBCommunity.org, Sydney, Australia; UP Manila: University of the Philippines Manila, PHILIPPINES

## Abstract

People living with chronic hepatitis B (PLCHB) are recommended to follow a lifelong monitoring regimen and face increased risk of liver cancer. Additionally, PLCHB frequently encounter stigma and discrimination, and relationship disruptions because of their chronic hepatitis B (CHB). Social support plays a key role in coping with chronic illnesses; however, this is inadequately assessed for PLCHB. This study aims to assess the physical, social, and mental impacts of living with CHB, the strategies PLCHB utilize to cope with their disease, and how social support–or lack of–impacts their journey with hepatitis B. The study was promoted through the Hepatitis B Foundation social media platforms, interested individuals filled-in a form expressing their interest to participate. The researcher conducted 24 telephone interviews in English, with PLCHB ≥18 years of age residing in the United States (U.S.) and Canada. Questions focused on the lived experiences of CHB and explored social support mechanisms that helped PLCHB. PLCHB experience a wide range of impacts (physical, social, and mental) that negatively affect their quality of life. Participants reported that receiving social support from their close network of individuals, hepatitis B community, or healthcare providers positively influenced their perspective on their future health and helped them adhere to treatment. The physical, social, and mental impacts of living with hepatitis B significantly affect the quality of life of PLCHB, calling for more research to document these impacts, and design integrated care models to address them. Social support appears to play an essential role in helping PLCHB cope with their disease and should be further studied.

## 1. Introduction

CHB is considered clinically silent for much of the infection, but PLCHB still experience impacts during this so-called asymptomatic phase [1]. The widely accepted biomedical framework is illustrated by management guidelines that focus only on clinical endpoints [2–5]. Such a framework de-prioritizes other care (non-clinical) needs of PLCHB (e.g., managing fear of disease progression, stigma, managing anxiety around the possibility of infecting others, knowledge about the disease, and lifestyle modifications) [6]. Given that CHB can quickly change in its trajectory (e.g., sudden development of symptomatic and severe liver disease), PLCHB experience the burden of constant lifelong monitoring [2,3,4,7,8], coupled with high levels of uncertainty over the course of the disease and the risk of liver cancer [9,10].

Recent studies focusing on Health-Related Quality of Life (HRQoL) have shown that PLCHB face physical, emotional, and mental symptoms that negatively impact their quality of life [11–13]. Physical symptoms included fatigue and muscle pain [11,12,14]. Emotional and mental symptoms included anxiety about transmitting the infection to others, and fear of developing liver cancer [9–13]. PLCHB also consistently report experiencing discrimination and stigma [11,12,15], including self-stigma, and hesitate to share their hepatitis B status with family, friends, colleagues, and care providers for fear of facing repercussions [10–13,16].

Social support can improve HRQoL in people with other chronic conditions [17] but its impact on the lived experience of CHB remains largely unexplored. While social support has a variety of definitions [18], we used the most relevant for our study: “the perception or experience that one is loved and cared for by others, esteemed and valued, and part of a social network of mutual assistance and obligations.” [19]. Social support may take different forms, like providing information, knowledge, and guidance [20], providing emotional support through love, affection, empathy, or providing the sense of affiliation and social integration [20,21]. Social support enhances sense of belonging and affiliation and empowers individuals to take active steps to face the challenges related to their illness [20,21]. In our study, we posit that social support may improve HRQoL reported by people with CHB, and their adherence to hepatitis B management, including adherence to healthy lifestyle behaviors (e.g. eating balanced healthy food and abstaining from drinking alcohol) and regular monitoring.

In this qualitative study, we explored the physical, social, and mental impacts of living with CHB, and how they affect their quality of life, and the role of social support in helping PLCHB cope with their disease and enabling them to face the challenges related to CHB.

## 2. Materials and methods

### Ethics statement

All methods were carried out in accordance with relevant guidelines and regulations. The study that included collecting this data was approved by Heartland Institutional Review Board LLC (HIRB Project No. 191221–270). Participants signed an informed consent prior to participation in the study. Participants’ consent was also confirmed verbally prior to starting the audio-recorded interview.

Data for this study were collected via in-depth telephone interviews. Potential interview participants were recruited between January 2020 to April 2020, via purposive sampling to ensure that we reached those living with CHB. The opportunity to participate was sent to potential participants via organizational e-newsletters, social media posts (reaching ~45,000), and a link at the end of an online survey about the same topic [22].

To be eligible for participation, individuals had to be at least 18 years old, have been diagnosed with CHB (self-reported), live in U.S. or Canada, and be able to communicate in English. Interviewees signed informed consent prior to the interview. A qualitative, semi-structured interview guide was developed and reviewed by a panel of experts and a PLCHB (S1 Appendix). Interview questions focused on the impact of living with CHB on relationships, career, and personal experiences managing CHB. Questions also probed social support and coping mechanisms that PLCHB utilized to cope with their disease. No personal identifying information was collected from interview participants during or after the interviews. Each interview lasted 60–90 minutes.

A total of 24 telephone interviews were conducted, recorded, and transcribed between February 2020 and May 2020. The resulting data were analyzed using a directed content analysis approach, creating a codebook to guide the organization of content “codes” or themes (S2 Appendix). Each code was clearly defined to ensure coding accuracy and improve inter-coder reliability. A team of five trained researchers worked on coding the interviews, using Dedoose qualitative analysis software (version 8.3.45) [23]. Each interview transcript was double-coded independently by two trained research team members to ensure coding accuracy. Inter-coder reliability (ICR) was assessed using Dedoose ICR calculation tool for each interview (ICR ranged from 65%-75%). Both coders reviewed discrepancies in an open discussion until an agreement was reached. The coding team met regularly to discuss emerging codes and organize themes.

## 3. Results

### A. Demographics

There were 24 interview participants from the U.S. and Canada. Most were 31–50 years of age (Table 1). Racial representation reflected the burden of HBV disease in North America, with 63% representing communities of color. Gender identity was split equally (50% female, 50% male). The majority (63%) of participants had been diagnosed for over ten years. Most (62%) were taking antiviral treatment for hepatitis B at the time of the interview.

**Table 1:** Demographics, years since diagnosis, and treatment experience of interview participants. (N = 24).

	Number (%)
Average Age	
18–30 years old	3 (12.5%)
31–50 years old	12 (50%)
51–70 years old	9 (37%)
Gender (female)	12 (50%)
Race	
White/ Caucasian	9 (37%)
Black/ African/ African American	4 (17%)
Asian/ Asian American	11 (46%)
Years since diagnosis	
0–5 years	3 (12%)
6–10 years	6 (25%)
> 10 years	15 (63%)
Currently on antivirals	15 (62%)

### 3.B. Themes

Interview participants described physical challenges, the social impact of living with CHB, and fears and worries derived from their hepatitis B status. They also described strategies they used to cope with their disease, including the critical role of social support.

It is notable to mention that the physical, social, and mental impacts of living with CHB were comparably reported across race and gender of interview participants. Likewise, the high emotional toll of their lived experiences with CHB was equally reported across the different number of years since diagnosis.

#### 3.B.i. Physical impact

CHB has affected interviewees’ ability to maintain their daily activities, including activities they considered important for them. Interviewees described frequent episodes of fatigue, pain, tiredness, and inability to finish their tasks at work and home, engage in activities they had enjoyed in the past, or participate in social activities. “… *The fatigue and under pain*, *it just got to a point where I just couldn’t deal with it anymore; it was hard to control*. *And with that also came my inability to work*. *So*, *I had to resign from my job; I couldn’t perform my job anymore*” [African American male, 39 y/o]. This interviewee also added; *“I also love to play sports*. *I haven’t been able to do that since 2015”*. Some have also attributed missing social events to their lack of energy, “*I needed more rest*. *I’ve had to curtail a lot of what I do*. *Especially with my kids’ activities”* [Asian female, 57 y/o] *“My husband was great*, *he used to take them all to all their early morning practices and events*”; the same interviewee explained how her husband was able to help with this situation. Interview participants occasionally reported these episodes to their medical providers. They were often dismissed: “*when I went to my doctor*, *I complained about this ‘extreme fatigue*,*’ and he said*, *‘It’s probably depression or maybe your chronic fatigue or whatever*.*’ I said*, *‘I know my body*.*’ Which I’ve always been accurate with*, *with anything I have*. *I said*, *‘Trust me*, *this isn’t depression*,*’*” [Caucasian male, 70 y/o]. Another described: “*I had been tired*, *but the doctors had always said that there was nothing [wrong]–my viral levels didn’t indicate the fact that I should be feeling any symptoms*" [Caucasian female, 34 y/o]. This dismissal of the physical experience by their medical providers was described as a source of disappointment.

In addition to the pain and lack of energy, several interviewees mentioned that they suffered from a lack of focus and fogginess. This symptom impacted their personal lives and careers: “*I know my brain function isn’t close to what it used to be*. *But it’s good enough to remember my doctor’s appointments*, *[and] it’s good enough to communicate and to even do hard processes*. *But I know I couldn’t sit and do what I used to do*,” [Caucasian male, 50 y/o].

The physical impact of living with CHB is significant. It has serious implications to one’s daily activities and social life, forcing PLCHB to abandon physical activities and social events, and leading to reduced quality of life.

#### 3.B. ii. Social implications

CHB is a highly stigmatized disease, and those living with CHB repeatedly report experiences of stigma, discrimination and feeling isolated. Interview participants mentioned experiences impacting their social lives following their diagnosis of hepatitis B. Many felt they had to self-isolate or avoid being with others for fear of being shamed, stigmatized, or discriminated against if others knew their hepatitis B status. One individual stated, “*I don’t know if I can put it in words*, *but the feeling of you isolating yourself from everything that you do for fear of what might happen if someone gets to know [my hepatitis B status]…*.” [African American male, 39 y/o]. This isolation and fear led many PLCHB to keep their hepatitis B status confidential: *“I didn’t disclose my condition*. *I lived with a secret*, *very deep down inside*. *So*, *on the outside*, *I appear perfectly normal*. *If I disclose [my hepatitis B status]*, *I may get stigmatized*, *who knows*, *but I’ve never revealed the fact that I have it”* [Asian female, 22 y/o].

The fear of being shamed or stigmatized because of hepatitis B deterred participants from seeking or finding support. A participant shared, *“I feel very alone about this*. *I don’t know anybody else that has hepatitis B*. *So*, *it’s not like there’s a bunch of people with diabetes or a bunch of people with some other chronic disease*. *I just feel very alone with this*. *There isn’t anybody that I know that talks about it because it’s such a stigma”* [Caucasian female, 44 y/o].

The widespread hepatitis B-related stigma; caused mainly by lack of awareness and misinformation about hepatitis B, has led PLCHB to feel socially rejected and compelled them to self-isolate. This has negatively affected their social well-being and consequently their quality of life.

#### 3.B.iii. Anxiety (mental impact)

The constant fear of disease progression and complications poses persistent stress and negatively affects the mental well-being of those living with CHB. Interview participants persistently expressed their fear of dying prematurely, leaving loved ones (mainly children) behind: “*My family doctor was telling me that I have [a] 25% [chance] of getting liver cancer*. *So*, *I worry about myself*. *However*, *with a child*, *I feel like this is important for me to carry through my motherhood*, *to raise the child*” [Asian American female, 60 y/o]. While fear of dying prematurely was mentioned as a source of anxiety for many participants, it was also discussed as a motivator for staying healthy and maintaining regular check-ups. One participant said: “W*hat can I do to bring myself having a lower risk of liver cancer so I can see my child growing up*, *being a good mom*” [Asian American female, 56 y/o]. Another described, “*I told the doctor; my goal is to see my son graduate high school and be able to take care of himself*. *I don’t think a child should have to go through a parent dying*. *So*, *I try to do everything possible that can help prolong my life*” [Caucasian male, 41 y/o].

Participants also expressed anxiety about the risk of transmitting the disease to others, especially to their children or partners. *“That’s my main worry [transmitting hep B infection]*. *When you have a partner*, *even if they’re vaccinated*, *how is it going to work*?*”* [African male, 41 y/o].

The continued anxiety about dying prematurely and/or transmitting the infection to others has led to significant harmful impacts on the mental well-being and the quality of life of PLCHB. Additionally, these fears have further impeded social interactions of PLCHB with their networks at different levels, which may have intensified their declined quality of life.

#### 3.B. iv. Coping mechanisms and social support

Several participants asserted that the support they received had a positive impact on how they could cope with the immense physical, social, and mental burden they face because of their hepatitis B status. Participants mentioned several forms of support that could be categorized into (1) a close circle of family, friends, and partners/ spouses; (2) being part of a community of people going through similar experiences (i.e. an organized network of PLCHB); and (3) healthcare providers.

#### 3.B.v. The role of family, friends, and partners

Family, friends, and partners played a crucial role in providing emotional support and care and sharing the burden. This has helped them endure the burdens of living with CHB and given them strength to maintain their well-being. One interviewee said, “*My family has been great; my close friends and my whole [family]–including my husband’s side of the family*, *they all know*. *And if anything*, *there is more an element of concern for me than there is judgment or stigmatism*” [Asian female, 57 y/o]. Another interviewee said: “*My wife*, *this is something we discuss pretty much anytime they do my ultrasound*, *or my blood work every six months and she would look at the result*, *and we sit down and discuss the different test result that comes out*” [African American male, 41 y/o]. “*The people I share with are my two sisters; both have medical backgrounds*. *So*, *all three of us have a common language and a common experience [with my father’s liver cirrhosis] and we share a lot*” [Asian American female, 56 y/o].

#### 3.B.vi. The role of community

Participants explained the importance of having a community to provide information and support. Participants referred to that community as an organized network of PLCHB and their caregivers. Such communities, though hard to find for some, were an effective source of support and provided individuals with knowledge and information that was often inaccessible even through healthcare providers. An interviewee described: “*Within a couple of years [from diagnosis]*, *I joined the [hepatitis B] support list*. *In the early years that was really very helpful emotionally*. *In terms of information*, *people were sharing what they knew*, *and that just sort of opened this whole world of available information*. *And I ended up going to the patient conferences*, *where there were panel discussions with physicians; very interesting and very helpful*” [Caucasian female, 64 y/o]. Another participant described how knowledge has empowered him: *“With my involvement in hepatitis B education in the community*, *this broadened my knowledge on the subject*. *It helped me improve my knowledge and then my wisdom about hepatitis B*. *So*, *I’m more comfortable now than I was when I was diagnosed”* [African American male, 39 y/o].

The community interaction also offered a strong sense of solidarity with others who shared similar experiences and were able to empathize with the difficulties and challenges through first-hand knowledge. One participant described their experience with a community listserv: “*When I could read other people’s [experiences]*, *that some of their experiences mirrored mine*, *just hearing of some of them going through any pain*, *or digestive issues*, *or the fatigue*, *or the headaches*, *or the insomnia*. *Seeing those things gave me some solidarity*, *unity*, *something that I couldn’t get anywhere else—even my girlfriend couldn’t give me that type of comfort because she never experienced it*” [Caucasian male, 50 y/o]. Another said, “*I found [on HBV list] a very good friend—sometimes I think we share our anxiety and emotion*. *We talk*, *and she has been very helpful*, *and it is mutual support*” [African male, 41 y/o].

#### 3.B.vii. The role of healthcare providers

Many participants discussed the impact of a strong relationship with their doctors, noting their support throughout diagnosis and management. Healthcare providers were repeatedly cited as a reliable source of information and a source of emotional support through assurance and transparent discussions about treatment options and disease perspectives. One interviewee said that her relationship with her doctor helped her through her maternity journey while living with CHB, “*Fortunately*, *I had very good support from my doctor and my gynecologist*. *I gave birth to two healthy children*” [Asian American female, 56 y/o]. Another explained how his doctor takes the time to explain to him what he needs to understand: “*I pretty much understand it because I have a pretty good gastro[enterologist]*. *He explained it to me well*, *and he researches*. *He’s been very supportive*, *and [he] has a good bedside manner*” [Asian American male, 39 y/o].

In addition to physicians’ role as healthcare providers, interviewees described looking to their physicians as a trusted source of information and for assurance. Those individuals who described lack of provider support, reported feelings of anxiety from the unknown and feeling vulnerable, and described their providers as not caring about their health and wellbeing. One participant mentioned that when he was diagnosed, his healthcare provider did not explain to him what hepatitis B was or what his diagnosis meant for him. He explained that this has left him fearful of a disease he had little information about. “*It’s so difficult because to me when I found out about my diagnosis*, *they just told me*, *‘You have hepatitis B*.*’ That was it*. *They didn’t offer any counseling; they didn’t tell me anything else*. *They just said you have it and that was the end of the case*. *They gave me a copy of the result and that was it*. *So*, *I had to figure out*, *okay*, *now I have this diagnosis*, *what is even hepatitis B*, *and try to educate myself on it*. *And once I started doing my own research and learning more about it*, *it answered some of the questions that I had that was tough for me to answer*” [African American male, 39 y/o].

## 4. Discussion

This study highlights the physical, social, and mental impacts of living with CHB on the quality of life of PLCHB, and the positive role that social support can play in coping with the disease. Study participants have reported significant physical, social, and mental effects of living with CHB that had negative bearings on their lives. They have also explained the role of social support–or lack of–in the effectiveness of coping with their disease.

The impact of CHB on HRQoL has been documented through both quantitative and qualitative studies [9,10,12–14,24,25]. However, HBV management guidelines focus on clinical, laboratory, and imaging endpoints, leaving out physical, social, and mental impacts that are meaningful to those living with the disease. This remains an unmet need for PLCHB [2–5,26]. Therefore, clinicians, scientists, patients, and regulators should come together and discuss effective ways to incorporate regular assessment of HRQoL of PLCHB, not only to evaluate their physical and mental health status, but also to identify their needs and develop strategies and programs to provide them with much needed resources and support.

Our results show that PLCHB face significant physical, social, and emotional burdens because of their hepatitis B, which further negatively affects their social integration into the communities they live within and poses a risk to their health outcomes and quality of life. However, our study shows that those with perceived social support report feeling empowered and capable of facing the challenges associated with CHB. Previous studies support our findings, showing that social support enhances a sense of community, boosts satisfaction with life, and is associated with positive rating of health and health outcomes [27,28]. Others have found that social support is critical for education and provision of information [29], and is associated with improved use of healthcare services, especially among immigrant populations [28], and better adherence to treatment [17]. Social support also enables navigation of the disease journey, and positively impacts physical functioning, cognitive function, and emotional health [28–31].

The role of family, friends, and partners was reportedly vital for participants to cope with their disease and set their goals of adherence to regular monitoring and treatment. The feeling of being loved and cared for, and having someone to rely on, provided relief from anxiety and uncertainty. This has also been reported by similar studies involving people living with HIV [30,31], people living with liver disease [32], those with chronic illnesses in general [17], and even among the general population [21]. These results highlight the importance of programs that educate and enable caregivers to support their loved ones through their lifelong journey with CHB.

Healthcare providers are key in educating, assuring, and guiding their patients to appropriate management and treatment options. Their role is essential to provide their patients with information related to hepatitis B, its symptoms, and how to manage these symptoms. Additionally, referrals to peer support groups, counselling services, or mental health services are instrumental in helping their patients cope with their disease. In our study, participants who reported a positive relationship with their physicians, supporting them with knowledge and guidance, took an active role in managing their CHB. Previous studies have shown that physicians play an important role in educating their patients, supporting their decision-making, and enhancing self-care [33–35].

Impacts of living with CHB were similarly reported across race and gender, which suggests that the human response to CHB transcends specific cultures (though the framing, response, and degree of impact may be varied). It also underscores the need for well-designed and structured social support networks that should be widely available and accessible.

Research exploring social support and coping mechanisms among PLCHB is scarce, but our work highlights its potential in improving HRQoL for PLCHB. We need to better understand the role of community support and invest in the creation of safe spaces (such as support groups) for PLCHB to exchange knowledge and experiences. Additionally, there is a need to explore strategies for integrating social support services into the care cascade and clinical management for PLCHB and their caregivers.

## 5. Limitations

This study had some limitations. The sample included only those living in the U.S. and Canada, which meant that participants may experience better access to care, supportive community-based organizations, and more access to knowledge and information about CHB when compared with other nations. Additionally, recruitment and data collection were conducted in English only. The majority of PLCHB living in U.S. do not speak English as their primary language [36], and future studies should incorporate multi-language data collection, and collaboration with researchers from other parts of the world to amplify the voices of those who are not English speakers.

## 6. Conclusion

The physical, social, and mental impacts of living with hepatitis B significantly affect the quality of life of PLCHB, calling for more research to document these impacts, and design integrated care models to address them. Social support can play an important role for PLCHB, and more studies are needed to explore this, and to consider ways to improve social support for PLCHB worldwide. Strategies should engage healthcare providers, families and caregivers, and patient organizations. Ultimately, this will improve the health and wellbeing of PLCHB.

## Supporting information

S1 Appendix Interview guide. (PDF)

S2 Appendix Interview analysis code. (PDF)
