# Supplementary material for: Quality of life of people living with chronic hepatitis B: The role of social support system
Source: PLOS Glob Public Health. 2024 Apr 26;4(4):e0003103. doi: 10.1371/journal.pgph.0003103 (PMC11051608; doi:10.1371/journal.pgph.0003103)
Supplement: S1 Appendix — (PDF) [file pgph.0003103.s001.pdf]

HEPATITIS B FOUNDATION
3805 Old Easton Road
Doylestown, PA 18902
215-489-4900 • hepb.org

Hepatitis B Foundation Patient Engagement Interview Guide

Welcome Remarks (5 minutes)
Thank you for having this call with me today, and for verbally consenting to participate in this project. My name is Yasmin, and I work with the Hepatitis B Foundation. The Hepatitis B Foundation is a national nonprofit organization that was started in 1991 with the mission of finding a cure for hepatitis B and improving the lives of those affected worldwide through research, outreach and patient advocacy. The Foundation website reaches 2 million people each year and counselors respond every year to almost 10,000 people for information and support through email, social media, and telephone calls. It also supports public health research and basic scientific research with 50 scientists working on a cure.

I would like to get your thoughts and opinions about living with chronic hepatitis B, and the future of hepatitis B treatment. Your thoughts are very important for us to help develop new medications and new programs for people with hepatitis B.

Before we begin, I'd like to let you know that during this discussion, there are no right or wrong answers, we just want to know your honest thoughts and opinions. This discussion will be recorded, so that we can accurately incorporate your feedback. You will remain anonymous, and your name will not be collected or identified. All discussion today will be kept confidential.

I would also like to confirm that your participation is voluntary and that you are not obliged to finish this telephone interview.

You will be compensated for your time today with a $20 e-gift card that you will receive via email after the interview.

Do you have any questions before we begin?

Let's Get Started.

Topic I. Patient Journey from Diagnosis to Living with Chronic Hepatitis B (25 minutes)

I would like to start at the beginning to understand the experience of living with chronic hepatitis B from your point of view. First, please think back to when you were diagnosed with <u>chronic</u> hepatitis B. Could you briefly share with us what led up to your diagnosis?

1. Can you tell us how long ago you were diagnosed with chronic hepatitis B?

2. Can you describe any physical symptoms that caused you to get tested or that you experience with your chronic hepatitis B?
   Discussion prompts: fatigue, joint pain, right-sided stomach pain, "brain fog," etc.
   a. What symptoms are the most bothersome to you and how often do you have them?
   b. If you have (or had) physical symptoms, how have they affected your life?

3. What impact has your diagnosis had on your family and those close to you?
   a. How has it affected your family relationships? Your social relationships?

4. What impact, if any, has your diagnosis had on your professional life?
   a. How has it affected your career path or your relationships with co-workers?

5. Can you share with us the emotional impact of living with chronic hepatitis B?
   Discussion prompts: shame, stigma, guilt, depression, anger, frustration, isolation, fear
   a. Have you changed any behaviors or daily activities as a result of your feelings?
   b. Can you tell us how you cope with the emotional impact of living with chronic hepatitis B?

6. Have you ever felt stigma or shame about your diagnosis? (Ask if not covered above)
   Discussion prompts:
   a. Have these feelings changed your behavior or daily activities in any way?

7. Have you ever felt discriminated against because of your hepatitis B?
   Discussion prompts:
   a. How has this experience affected your life or daily activities?

8. Are you able to share your feelings about living with chronic hepatitis B with other people?
   Discussion prompts:
   - If yes, who are you sharing with?
   - If not, what makes it difficult for you to share this information with others?

9. What, if any, concerns do you have about transmitting hepatitis B to others?
   Discussion prompts:
   a. How has this impacted your daily life?

Topic II. Patient Experiences on Current Treatments for CHB (10 minutes)

Let’s talk about your thoughts about the current treatment of hepatitis B.

10. Have you ever been treated for hepatitis B with an approved treatment prescribed by a doctor?

    Discussion prompts:

    a. If YES, how long have you been taking hepatitis B medication and what is the name?
        i. If it was in the past, how long were you treated and what was the name?
    b. If NO, what are your reasons for not taking medication?
        i. What would need to change (or happen) for you to start treatment?

11. Can you tell us whether you feel your current treatment is making a difference in managing your chronic hepatitis B?

    Discussion prompts:

    a. How do you feel about being on treatment?
    b. How has treatment impacted your life?

12. Can you tell us if there are any challenges to taking your medication every day?

    Discussion prompts:

    a. Do you experience side effects with your current medication?
    b. Are there any potential long-term effects that you worry about with your treatment? (for example, osteoporosis or kidney damage)
    c. Is cost a challenge?
    d. Is access a challenge? (for example, can’t get to the pharmacy; complicated specialty pharmacy or mail order rules, etc.)

13. Have you ever missed doses of your daily medication and if yes, what are some of the reasons?

    Discussion prompts:

    a. Too busy and I forget
    b. Don’t want to be reminded of my chronic condition
    c. It’s expensive so I skip doses to make my pills last longer (ration pills)
    d. It’s hard to get my pills every month so I miss doses (access issues: transportation, access, specialty pharmacy and mail order rules)

TOPIC III - Patient Perspectives on an “Ideal Treatment” for CHB (30 minutes)

So now we want to ask about your thoughts about future treatments.

The currently approved treatments for hepatitis B are not cures, but they manage the disease by suppressing the virus (e.g. decrease viral load) to undetectable levels in the blood; thereby, "slowing down" liver damage. These drugs, however, must be taken for many years (possibly even a lifetime) and they don't completely eliminate the virus from the liver, so there is still a risk of developing liver cancer later in life.

14. If you were sitting in a room full of people who are working to create treatments for chronic hepatitis B, what 3 things would you tell them are the most important issues that they should address for an "ideal treatment"? Why?

    Discussion prompts:

    a. Being able to stop taking my medication after 6-12 months
    b. Experiencing an improved quality of daily life (less fatigue, joint pain, "brain fog")
    c. Loss of hepatitis B surface antigen (testing negative for HBsAg)
    d. Sustained or long-term undetectable viral DNA (no viral load)
    e. Decreased risk of developing liver cancer

15. What do you feel would be the key benefit of an "ideal treatment"?

An important question that we want to talk about is <u>treatment terminology</u> for the new hepatitis B drugs that are already in human clinical trials. I'm going to show you flash cards that describe two different treatment outcomes for chronic hepatitis B, which we'll then discuss.

"Complete" vs. "Functional" Cure Flash Cards. Please take a moment to read the information in the card you have.

<u>Treatment Outcome #1 - A "Complete Cure"</u>
Researchers define a "Complete Cure" for hepatitis B as being the loss of cccDNA, which is the covalently closed circular DNA that is found inside the nucleus of the hepatitis B virus. The cccDNA usually persists inside liver cells for very long time. If a drug could eliminate the cccDNA, then the hepatitis B virus can no longer reproduce and a "complete cure" would be achieved. But, as long as there is cccDNA, the hepatitis B virus cannot be completely eliminated from the liver; and thus, a person has a continued risk of progressive liver damage, potentially leading to liver cancer. Currently, there are no drugs that can eliminate cccDNA.

<u>Treatment Outcome #2 - A "Functional Cure"</u>
New drugs in the research pipeline show promise of what researchers call a "Functional Cure." That is, the new drugs result in the loss of the hepatitis B surface antigen (HBsAg), which means there is no detectable virus in the blood. This can occur with or without development of antibodies against the hepatitis B virus (HBsAb+ or anti-HBs+). A "functional cure" does not eliminate cccDNA, but it will maintain long term suppression of the viral DNA and reduce the risk of liver cancer even when treatment is stopped. Most importantly, a "functional cure" would result in new drugs that would be taken for a limited time (possibly up to one year) rather than a lifetime.

16. Most people will not have heard this information before. We want to know what your reaction is to this information.

    Discussion prompts:

a. What does this mean for you as a patient?

17. How relevant would you say a "functional cure" is for you?

Discussion prompts:

a. What difference if any would it make to you?

18. Do you think the terminology 'functional cure' is appropriate and meaningful? Why or why not?

19. I'm going to present some alternative terms for you to consider instead of "functional cure." Please tell me if any of the terms are more meaningful to you? (Read them first, and then repeat each term if necessary)

- Partial Cure
- Remission
- Sustained Viral Response
- No preference
- Other

Topic IV - Patient Perspectives on Clinical Trials for CHB (15 minutes)

Let's talk about the new drugs in the research pipeline that work differently from the current hepatitis B drugs. The key difference is that many of the new drugs result in the loss of hepatitis B surface antigen (HBsAg), which means there is no detectable virus in the blood. In addition, these new drugs would only

have to be taken for a limited amount of time (possibly up to 1 year). And once treatment was stopped, there would still be long term suppression of viral DNA and a significantly decreased risk of liver cancer.

20. With many promising new drugs on the near horizon, would you be willing to participate in a clinical trial for a new hepatitis B drug?
    Discussion prompts:
    a. What would make you willing TO participate?
        i. Doctor recommends, high/moderate chance of positive outcomes, free medical care, easy to get to, helps others, and advances research
    b. What would make you NOT willing to participate?
        i. Dr. doesn't recommend, worried about changing drugs, fearful of study process, bad personal experience or someone I know had a bad experience

21. If there was a new treatment for hepatitis B that met your criteria for an "ideal treatment," can you tell us what would you be willing to accept in terms of how the drug is given, and how long it would have to be taken?
    Discussion prompts: (Route of administration and duration)
    a. Would you be willing to have a weekly 1-hour intravenous infusion (IV) for 3 – 6 months?
    b. Would you be willing to have a weekly subcutaneous injection 6 – 12 months? (this is an injection given under the skin, not the muscle)
    c. Would you be willing to take a pill every day for 6-12 months?
    d. Would you be willing to take a combination treatment with a daily pill and a weekly IV or subcutaneous injection treatment for 6 – 12 months?

22. Can you tell us what would you be willing to accept in terms of side effects?
    Discussion prompts: (Side effects)
    a. Would you be willing to have temporary mild side effects like headache, mild nausea?
    b. Would you be willing to have mild to moderate flu-like symptoms or fatigue for a couple days?
    c. What side effects would be unacceptable?

23. What percentage of success to achieve an "ideal treatment" outcome would be required for you to consider participating in a clinical trial for hepatitis B? For example, 25%, 50% … or more?

Contingency Questions: (Only ask if the focus group is running early or on time)

- What do you think pharmaceutical companies could do to improve clinical trial participation?
  Discussion prompts:

    a. Although the FDA does not allow companies to pay people to participate in a clinical study, there could be alternatives like reimbursing travel, childcare, lost wages. What kind of support would make it easier or more likely for you to participate?

- What is your understanding of your diagnosis right now? Are there any areas that you are not sure about or still wish you understood better?

Before we end our discussion, are there any final thoughts or questions that people want to share about the topics we discussed?

Thank you very much for your time today, we sincerely appreciate you sharing your thoughts and questions with us to better understand what it is to live with chronic hepatitis B.

We will now end the discussion and stop recording.
