# Supplementary material for: Quality of life of people living with chronic hepatitis B: The role of social support system
Source: PLOS Glob Public Health. 2024 Apr 26;4(4):e0003103. doi: 10.1371/journal.pgph.0003103 (PMC11051608; doi:10.1371/journal.pgph.0003103)
Supplement: S2 Appendix — (PDF) [file pgph.0003103.s002.pdf]

Codebook:

1. **Diagnosis** (use when individual is describing their HBV diagnosis)
    1. **Lifestyle modification** (use when individual describes any changes positive or negative made as a result of hepatitis B diagnosis)
2. **Emotional Impact of HBV Diagnosis** (use when individual is describing the emotional feelings/descriptors related to their HBV diagnosis or living with hepatitis B ex: emotional feelings/anger/concerns/challenges associated with HBV)
    a. **Fears/ Concerns** (use when individuals express feeling of fear/ concerns about future events or how their disease progression might affect their health/ life trajectory, for example fear of developing liver cancer, or fear of dying and leaving children behind)
3. **Physical impact/symptoms** (use when an individual describes physical challenges associated with living with hepatitis B ex: pain, discomfort, etc.)
    a. **HBV Progression** (use when individuals discuss long-term consequences of HBV indicating decompensation, ex: precancerous lesions, fibrosis, cirrhosis, liver cancer or liver transplant)
4. **Sharing Hepatitis B Status w/others** (Use when individual is describing if/how they have talked about their HBV status with others (personal or professional) and its impact)
5. **Stigma** (use when individual is describing the stigma related to HBV they have experienced in their communities internally (internalized-stigma/self-stigma) or externally (within community context) within social contexts)
6. **Discrimination** (use when individual is describing the discrimination related to HBV they have experienced personally, i.e. institution discrimination)
7. **Support/ Coping Mechanisms** (use when individual describes mechanisms that help them cope with challenges associated with living with CHB, e.g. going to church, family support, supportive partner)
8. **Barriers** (use when individual describes challenges associated with hepatitis B, this can be double coded with specific sub-codes below if individual describes within text)
    (a) **Forgoing Care** (use when individuals mention that they didn't seek medical care whether when they were first diagnosed or later in the course of their care). *Do not use if an individual stops care because of other barrier listed in the codes.*
    (b) **Medication Barriers** (Use when individual describes having challenges associated with accessing medication for HBV)
    (c) **Physician Barriers** (use when individual describes a challenge related to physician access, communication or finding a physician competent in HBV management)
        i. **Physician-patient Communication** (use when individuals describe challenges related to physicians' communication, explaining disease and treatment consequences, and listening to their patients' concerns)
    (d) **Cost Barriers** (use when individual describes challenges associated with cost burdens related to HBV)
    (e) **Pharmacy Barriers/Medication Access** (use when individual describes challenges associated with pharmacy and accessing their HBV medication)
9. **Medication/Treatment Experience** (Use when individual describes their current medication status related to HBV (positive or negative) and their current treatment

experience related to HBV (positive or negative), even if they are not currently on treatment)

(a) **HBV Management** (use when individuals discuss their management of their HBV, like regulars doctor visits, U/S and lab routine ... etc.)

(b) **Side Effects** (use when individual describes side-effects associated with being on treatment side effects). *Do not use if individual is discussing side effects related to HBV progression.*

10. **Future Medication Recommendations** (use when individual describes ideal or recommendations related to future medications for HBV)

    (a) **Functional Cure Feedback** (use when individual describes their thoughts on a functional cure)

    (b) **Future Treatment Preferences** (use when individual describes treatment duration/ route of administration preferences for HBV future therapy)

11. **Clinical Trials** (use when the individual is talking about or describing their current experience or future recommendations for clinical trials related to hepatitis B treatment)

12. **Areas of Knowledge Gaps** (Use when individuals discuss knowledge gaps about HBV among the public, or missing information they need to know/ learn about for their disease management)
